# Supplementary material for: Are carbon emissions trading and green financial instruments synergistic? -Comprehensive quantitative research based on content analysis
Source: PLoS One. 2024 Mar 7;19(3):e0298601. doi: 10.1371/journal.pone.0298601 (PMC10919601; doi:10.1371/journal.pone.0298601)
Supplement: S1 Data — (ZIP) [file pone.0298601.s001.zip › Data/地级市Z_F工作报告.zip!/╘╞─╧/└Ñ├≈╩╨/└┤╫╘╣1⁄2╓┌║┼í╛┬φ┐╦╩2╛▌═°í┐_2016.docx]

各位代表：

我代表市人民政府，向大会报告工作，请连同《昆明市国民经济和社会发展第十三个五年规划纲要（草案）》一并审议，并请各位政协委员提出意见。

一、2015年和“十二五”时期工作回顾

刚刚过去的2015年，面对错综复杂的国内外形势和艰巨繁重的改革发展稳定任务，我们在省委、省政府和市委的坚强领导下，在市人大及其常委会和市政协的监督支持下，团结带领全市各族人民，认真贯彻党的十八大和十八届三中、四中、五中全会精神，全面落实习近平总书记系列重要讲话和考察云南重要讲话精神，积极适应新常态，全力以赴稳增长、调结构、促改革、惠民生、防风险，实现经济社会平稳发展。初步预计，全市地区生产总值完成4000亿元、增长8%，其中，第一产业增加值增长6%，第二产业增加值增长7.5%，第三产业增加值增长9%。一般公共预算收入完成458.9亿元、增长4.1%。

一年来，我们重点推进了6个方面的工作：

（一）全力以赴稳增长。积极应对经济下行压力，认真贯彻落实国家、省稳增长政策，及时出台实施19条提振实体经济、24条促进房地产平稳健康发展等政策措施。激活项目增投资，争取到113.8亿元国家专项建设基金，规模以上固定资产投资完成2938亿元，增长10%。加大帮扶稳工业，投入工业稳增长资金3.7亿元，42个亿元以上工业项目竣工投产，规模以上工业增加值增长6.5%。挖掘潜力扩消费，社会消费品零售总额完成1937亿元、增长8%。

（二）加强城市建设管理。编制区域综合交通体系、城市地下空间利用等专项规划，中心城区控详规实现全覆盖。功东高速、武定至倘甸至寻甸高速开工，绕城高速东南段等项目加快推进，呈澄、功待高速公路建成通车。地铁4号线开工，3号线、1号线呈贡支线等工程顺利推进。新火车南站建设提速。扎实开展市容环境综合整治，通过国家卫生城市、节水型城市复审复查，滇池路等56条城市重点道路环境明显改善。

（三）深入推进改革开放。“三证合一”“一照一码”登记制度改革取得突破，新登记注册企业增长49.4%。市级政府部门权力清单、责任清单向社会公布。投融资、财税、教育、卫生、社保等领域改革稳步推进。高新区和腾俊国际陆港B型保税物流中心获得国家批准。昆蓉欧班列开通。第3届南博会暨第23届昆交会、国际旅交会成功举办。与越南岘港市缔结友城关系。滇中四州市实现通讯同城化。引进市外到位资金828亿元、实际利用外资21.9亿美元。实现进出口总额125亿美元。

（四）扎实推进“三农”工作。粮食生产保持稳定，蔬菜、花卉等特色产业稳步发展，55个农产品获云南名牌农产品称号。启动都市农庄建设10个。新增农民专业合作社300个、省市示范社26个。启动2件中型、7件小型水源工程建设，建成“五小水利”工程5万件，解决17万农村人口饮水安全问题。全面开展“挂包帮”“转走访”工作，减少建档立卡贫困人口6.8万人和边缘贫困人口7.6万人。

（五）持续改善生态环境。滇池治理通过年度国家考核，建成第十一污水处理厂，铺设主城排水管网48公里，建成王官、斗南一期、东大河一期等湖滨生态湿地，疏浚滇池淤泥200万立方米。开展“省市联动·绿化昆明·共建春城”义务植树活动，植树造林2.7万亩，主城区新增绿地732公顷。单位GDP能耗下降9%以上，超额完成年度节能减排目标任务。主城环境空气质量优良率达97.7%。

（六）努力办好民生实事。民生支出占一般公共预算支出的比重达73.7%。城镇、农村常住居民人均可支配收入分别达34111元、11454元，分别增长9%、10.5%。城镇新增就业11.1万人，城镇登记失业率为2.98%。企业退休人员养老金人均增资217元。基本建成保障性住房、棚户区改造住房3.3万套，完成保障性住房分配、租赁住房补贴发放1.18万套（户）。30多万户居民用上天然气。建设、改造便民菜市场222个。557座城市公厕免费向市民开放。加快社区居家养老服务中心建设，新增养老床位3470张。公办幼儿园和普惠性民办幼儿园在园幼儿占比达60%以上。新增优质中小学40所。完成3个乡镇卫生院、166个村卫生室标准化建设。新建及改扩建文化站4个、文化室46个，古滇文化名城一期等文化工程建成使用。成功举办市第五届运动会。深入开展“平安昆明”建设，严防严打暴力恐怖、金融诈骗等各类违法犯罪。汲取“3·04”火灾、东川“4·25”矿洞炮烟中毒事故教训，加大安全隐患排查治理力度，安全生产形势总体稳定。

各位代表！2015年我市各项工作取得了较好成绩，为收官“十二五”作出了积极贡献。回顾过去的五年，我们战胜了诸多困难和风险，经受住了严峻挑战和考验，走过了极不平凡的历程，通过全市人民的不懈努力和艰苦奋斗，春城大地发生了新的变化、焕发了新的活力，为在全省率先全面建成小康社会奠定了坚实基础。

过去的五年综合实力迈上新台阶。经济总量突破4000亿元，年均增长11.5%，人均生产总值超过6万元，年均增长10.3%。一般公共预算收入年均增长14.5%。规模以上固定资产投资年均增长20.9%。社会消费品零售总额年均增长14.2%。三次产业结构由2010年的5.8∶45.2∶49调整为4.8∶41.2∶54。全社会研究与试验发展经费投入与地区生产总值之比为1.75%，成为国家创新型试点城市。

过去的五年城乡建设迈出新步伐。中心城区面积达412平方公里，比2010年增加125平方公里，户籍人口城镇化率达61.6%。市级行政中心顺利搬迁，呈贡实现撤县设区。西北绕城、南连接线等一批交通干线建成通车，绕城高速内环实现闭合，“环线+射线”骨干路网基本形成；地铁1、2号线首期工程、6号线一期工程投入运营，昆明长水国际机场开通国内外航线276条，市民出行条件大为改善。建成清水海引水工程、寻甸木戛利中型水库等一批水利基础设施。通乡油路率和行政村公路通畅率均达100%，建成新农村和美丽乡村634个，城乡人居环境明显改善，荣获“国家卫生城市”称号。

过去的五年生态建设取得新成效。全面推进滇池治理“六大工程”，环湖截污干渠闭合贯通，牛栏江—滇池补水工程实现通水，建成湖滨生态湿地3600公顷，滇池水域面积增加11.51平方公里，实现了还水予湖、湖进人退。滇池水质由重度富营养转为中度富营养，滇池主要入湖河道水质基本消除劣Ⅴ类，草海水质基本达到Ⅴ类，外海水质基本达到Ⅳ类。阳宗海水质基本恢复到Ш类。主城建成区累计新增绿地5386公顷，城市绿地率达38.2%。完成营造林475万亩，森林覆盖率达50%。超额完成“十二五”节能减排目标任务。环境空气质量优良率保持在95%以上。荣获“国家森林城市”称号。

过去的五年改革开放取得新突破。商事制度改革成效明显，国资国企改革不断深化，全面完成农村集体土地所有权确权登记颁证，集体林权制度配套改革深入推进。小升初、异地中考改革顺利实施。公立医院改革等医药卫生体制改革稳步实施。投融资、社会治理等各项改革全面推进。累计引进市外到位资金5700亿元、实际利用外资90亿美元，累计实现进出口总额740亿美元。滇中新区经国务院批准，成为第十五个国家级新区，阳宗海风景名胜区升级为国家级旅游度假区。南博会、旅交会影响力不断提升。孟加拉国等7国在昆设立总领事馆，新增印度加尔各答等8个国际友城，南亚东南亚友城数位列全国第一，昆明的国际知名度和影响力得到提升。

过去的五年人民生活水平得到新提高。城镇、农村常住居民人均可支配收入年均分别增长12.1%和14.6%。城乡居民基本养老保险制度实现全覆盖，建立了统筹城乡居民的基本医疗保险制度和大病保险制度。建设各类保障性住房35.3万套，城镇住房保障覆盖率达28%。学前教育基本普及，义务教育均衡发展成效显著，优质普高学校学生占比由67.2%提升到72.5%，人均受教育年限从8.9年提高到10年。城市居民“15分钟医疗卫生服务圈”初步形成，居民平均期望寿命从76.5岁提高到77.9岁。在全国率先实施基层公共文化服务包，“两馆一站”全部免费开放。现行标准下累计减少农村贫困人口19.32万人，贫困发生率由2010年的11.2%下降到2015年的4.8%。人民群众幸福感、获得感进一步增强。

过去的五年社会治理取得新进步。“党委领导、政府负责、社会协同、公众参与、法治保障”的社会治理格局逐步形成，社会治安持续稳定。积极化解征地拆迁、企业改制、农民工讨薪等领域的矛盾纠纷，公共突发事件应急处理机制日趋完善。开通“市长微博”，完善“市长热线”，畅通信访渠道，积极回应群众诉求。完善食品药品安全责任体系，监管能力稳步提升。加强军民融合式发展，驻昆部队在地方建设中的作用进一步增强。支持工会、共青团、妇联等人民团体开展工作。民族宗教、防灾减灾、气象服务、史志档案、港澳台侨等工作取得新成绩。

过去的五年政府自身建设取得新进展。深入开展党的群众路线教育实践活动、“三严三实”和“忠诚干净担当”专题教育，着力打通联系服务群众“最后一公里”。严格落实中央“八项规定”精神，切实解决“四风”突出问题，有效促进政风转变，“三公”经费年均下降11.5%。坚持依法行政，落实重大事项公示和听证制度，建立重大行政决策合法性审查制度。自觉接受人大法律监督、工作监督和政协民主监督，认真办理人大代表建议和政协委员提案。提交地方法规草案38件，制定、修订政府规章36件、废止12件。加快政府职能转变，市级行政审批事项精简为74项。落实党风廉政建设主体责任和“一岗双责”，加大反腐败斗争力度，严肃查办了一批违法违纪案件。

各位代表！过去的五年我市经济社会发展取得的成效，是省委、省政府和市委坚强领导的结果，是市人大及其常委会和市政协监督支持的结果，是全市各族人民团结拼搏的结果。在此，我谨代表市人民政府，向全市广大干部群众，向各位人大代表和政协委员，向各民主党派、工商联、无党派人士和人民团体，向中央和省级驻昆单位、各兄弟州市、驻昆人民解放军和武警部队官兵，向所有关心、支持昆明发展的海内外朋友，表示崇高的敬意和衷心的感谢！

在充分肯定成绩的同时，我们也清醒地看到，我市经济社会发展中还存在许多困难和问题，主要表现在：一是经济总量不大，发展质量不优，发展速度不快，产业竞争力不强，结构调整缓慢。二是城市规划建设品质不高，缺乏统筹谋划，城市管理体制落后，市容环境脏乱差现象仍然存在，交通拥堵严重。三是城乡区域发展不平衡、不协调，重城市轻农村，农村基础设施薄弱、历史欠账多，脱贫攻坚任务艰巨。四是资源环境约束趋紧，滇池治理任重道远，节能减排压力较大。五是社会事业发展不均衡，社会治理水平不高，社会矛盾多发易发。六是政府自身建设和民主法制建设亟待加强，干部作风有待进一步改进。

各位代表！“十二五”规划确定的地区生产总值、固定资产投资等经济指标没有达到预期，究其原因，既有我们对经济发展形势复杂性认识不足，判断过于乐观，也有经济结构不合理、传统动力减弱的深层次原因，还有自然灾害频发、社会矛盾凸显等问题叠加交织，同时也暴露出我们适应“新常态”的能力不足，解放思想不够，发展格局不宽，破解困难和问题的办法不多，部分干部主动作为、敢于担当的精神不强等问题。对此，我们将高度重视，综合施策，下大力气解决好这些问题，努力保持经济社会持续健康发展。

二、“十三五”时期的主要目标和任务

“十三五”时期，是我市转方式调结构的关键期、优布局提品质的调整期、促改革增活力的攻坚期、补短板建小康的决胜期、新优势新动力的形成期。我市发展面临难得的重大机遇，世界多极化、经济全球化、文化多样化、社会信息化深入发展，新一轮科技革命和产业变革蓄势待发，我国在全球治理体系中的话语权逐步增强，有利于我市参与国际合作交流，加快建设区域性国际中心城市；国家“一带一路”、长江经济带等重大战略深入实施，昆明作为“一带一路”和长江经济带的重要支点、面向南亚东南亚开放的重要门户优势更加凸显，有利于我市充分利用国内外两种资源、两个市场，在更大空间和更广领域加快发展；滇中城市经济圈一体化发展和滇中新区开发建设上升为国家战略，有利于我市优化资源要素配置和生产力布局、提升城市辐射带动能力。近期，李纪恒书记到昆明考察调研，省委省政府召开了推动昆明市改革发展座谈会，要求研究制定若干政策措施，举全省之力支持昆明改革发展，为昆明未来发展确定了定位、明确了方向，为昆明跨越发展注入了强大动力。只要我们紧紧抓住难得的发展机遇，着力破解发展难题，厚植发展优势，就一定能够闯出一条跨越式发展的路子来。

“十三五”时期，我们要高举中国特色社会主义伟大旗帜，全面贯彻党的十八大和十八届三中、四中、五中全会精神，以邓小平理论、“三个代表”重要思想、科学发展观为指导，深入贯彻习近平总书记系列重要讲话和考察云南重要讲话精神，按照省委九届十二次全会、市委十届七次全会的安排部署，认真贯彻落实好省委省政府推动昆明市改革发展座谈会精神，坚持“五位一体”总体布局和“四个全面”战略布局，贯彻创新、协调、绿色、开放、共享五大发展理念，主动服务和融入国家战略，着力当龙头、促跨越、上水平、惠民生、奔小康，加快建设立足西南、面向全国、辐射南亚东南亚的区域性国际中心城市，当好云南经济社会发展的排头兵和火车头，在全省率先全面建成小康社会，为谱写好中国梦云南篇章作出应有的贡献。

“十三五”时期，我市经济社会发展的主要目标是：到2018年，实现现行标准下全市贫困人口全部脱贫，贫困县全部摘帽，区域性整体贫困得到解决，在全省率先全面建成小康社会；到2020年，在巩固全面建成小康社会基础上，全市经济社会发展迈上更高水平，区域性国际中心城市建设取得重大进展，与滇中新区的融合发展成效明显，全省最具活力的增长核心基本形成，昆明的发展更科学、社会更和谐、文化更繁荣、生态更文明、人民更幸福，为开启社会主义现代化建设新征程奠定坚实基础。主要预期目标建议为：地区生产总值年均增长9%左右，一般公共预算收入年均增长6%，规模以上固定资产投资年均增长13%，规模以上工业增加值年均增长9%，社会消费品零售总额年均增长10%，城镇常住居民人均可支配收入年均增长9%，农村常住居民人均可支配收入年均增长10%。

“十三五”时期，我们要突出八个方面的着力点：

（一）着力提升城市功能和品质。按照中央对城市工作“五统筹”的要求，坚持量水发展、以水定城，科学确定城市发展布局、结构和规模，构建“两核一极两区六廊”空间布局。强化规划统筹引领，构建城乡一体、“多规合一”的规划体系。建设完善路网、航空网、能源网、水网、互联网，构建有效支撑跨越发展的基础设施体系。推进“智慧城市”建设。坚持保护提升老城，打造一批历史文化街区和特色街区，彰显历史文化名城魅力。加快新区拓展，提升呈贡新区科教研发、交通枢纽、信息服务等核心功能，打造现代化科教创新新城。加快推进巫家坝、草海、东风广场等重点片区规划开发建设，不断提升城市品质。积极联动滇中新区融合发展，把滇中新区建设成为国际化高新产业新城，打造支撑昆明、带动全省跨越发展的重要增长极。

（二）着力打造昆明经济升级版。坚持转方式、调结构，加大产业优化升级力度，坚定走开放型、创新型和高端化、信息化、绿色化、集群化的发展新路子，做特一产、做大二产、做强三产，加快推动昆明产业迈向中高端。大力发展高原特色农业，走产出高效、产品安全、资源节约、环境友好的农业现代化道路。改造提升化工、冶金、非烟轻工、烟草及配套等传统产业，培育发展先进装备制造、生物医药、电子信息、新材料等新兴产业。突出发展生产性服务业，加快发展生活性服务业，培育壮大商贸物流、金融、旅游、文化创意、科技及信息服务、房地产、会展、健康服务等现代服务业。创建全国质量强市示范城市，发挥标准化引领作用、认证认可规范作用和品牌示范作用。培育创新发展新动力，增强产业核心竞争力，成为全省构建现代产业体系的先行区。

（三）着力建设创新型城市。突破制约发展的体制机制障碍，扎实推进经济、行政管理、社会事业和生态文明等各项改革，充分发挥在全省全面深化改革中的示范引领作用，为跨越发展释放更多红利。深入实施创新驱动战略，构建以企业为主体的创新体系，加强创新平台建设，优化配置创新资源，推进开放式创新，培养引进高层次创新型人才，全社会研究与试验发展经费投入与地区生产总值之比超过2.5%，达到全国平均水平，成为全省“大众创业、万众创新”示范区、西部重要创新型城市和辐射南亚东南亚的科技创新中心。

（四）着力建设面向南亚东南亚辐射中心核心区。主动融入中国—中南半岛、孟中印缅经济走廊，加强与长江经济带、京津冀地区、成渝经济区及泛珠三角等国内区域的交流合作，当好滇中城市经济圈发展的“火车头”。提升南博会、昆交会、农博会等会展影响力，打造面向南亚东南亚的区域性国际会展中心。高标准建设昆明综合保税区和国家保税物流中心。大力发展开放型经济，打造面向西南开放的区域性国际金融服务中心、信息中心、物流中心和外向型特色产业基地。坚持“引进来、走出去”并举，提升招商引资质量，支持有条件的企业对外开展投资、劳务合作、工程承包。

（五）着力推进文化繁荣发展。加强精神文明建设，提高市民文明素质和城市文明程度，争创全国文明城市。健全完善现代公共文化服务体系，丰富公共文化产品供给，繁荣群众文化活动，推进“五馆”建设，统筹建设多功能基层综合性文化服务中心。推动文化创意产业与旅游度假、会展博览、观光农业等产业融合发展，大力发展数字创意、云媒体服务等新型业态。加大历史文化名城保护力度，加强非物质文化遗产保护传承，彰显名镇名村风采。加快推进文化交流、文化传播、文化贸易，构建具有区域影响力的民族文化传播中心。丰富群众体育活动，提升竞技体育水平，打造国际高原体育基地品牌。

（六）着力加强生态文明建设。强力推进以滇池为重点的水环境综合整治，深入实施和完善“六大工程”，提升滇池流域污水收集处理、河道整治、湿地净化、水资源优化配置效能。坚持实施城镇污染源控制，继续完善环湖截污和片区截污系统，深入开展入湖支次沟渠综合整治，优化湖滨生态系统功能，加强面源污染治理，持续开展内源污染治理。到2020年，力争滇池外海水质稳定达到IV类，草海水质稳定达到V类，主要入湖河道水质稳定达到V类以上，湖体富营养化水平明显降低，蓝藻水华程度明显减轻，流域生态环境明显改善。加大阳宗海、牛栏江流域水环境保护治理。加快建设“海绵城市”。大力开展城乡园林绿化，深入实施植树造林、退耕还林、天然林保护工程，建成区绿化覆盖率保持在40%以上，全市森林覆盖率达52%以上，构建“三屏两区一带”生态安全屏障。加强大气污染防治，环境空气质量稳定达到国家二级标准以上。推进低碳循环发展，完成省下达的节能减排目标任务。

（七）着力推进城乡区域协调发展。坚持以人的城镇化为核心，提升城镇化发展质量，户籍人口城镇化率达70%。提升县域产业层次和支撑能力，加快县域基础设施建设，深入推进扩权强县、扩权强镇，发展壮大县域经济。全面完善农村道路、能源、通讯、水利等基础设施，加快提升农村教育、医疗卫生、文化等公共服务水平，深入开展农村生态环境、人居环境综合整治提升，建设美丽宜居乡村。加大对资源枯竭、产业衰退、生态严重退化等困难地区的支持力度。深入推进精准扶贫、精准脱贫，围绕“脱贫、摘帽、增收”三大目标，强化基础设施和公共服务，加快培育特色优势产业，不断增强贫困地区发展后劲。扎实开展“挂包帮、转走访”工作，健全贫困县考核、约束、退出机制，落实贫困县区党政一把手扶贫开发主体责任，确保2016年禄劝县脱贫摘帽，2017年寻甸县脱贫摘帽，2018年东川区脱贫摘帽，坚决打赢脱贫攻坚战。

（八）着力建设安居乐业的和谐昆明。坚持就业优先战略，完善就业政策、服务体系，鼓励创业带动就业，城镇登记失业率控制在4%以内。全面普及学前教育，促进县域义务教育均衡发展，普及高中阶段教育，支持民办教育、职业教育、特殊教育、高等教育和老年教育发展。建立覆盖城乡的基本医疗制度，提升公共卫生服务水平，构建多层次养老服务体系，推进健康昆明建设。实施全民参保计划，建立城乡一体的社会保障体系。健全社会救助机制，保障困难群体基本生活。落实粮食安全行政首长责任制，完善安全生产和食品药品监督管理体系。加强社会治安综合治理，提升公共安全保障水平。推进社会治理精细化，健全基层管理和服务体系，引导群众依法行使权利、表达诉求、解决纠纷。积极支持驻昆部队建设和军队改革，不断提高双拥共建水平。全面推进依法行政，着力建设法治政府。

三、2016年的工作

今年是全面实施“十三五”规划的第一年，开好局、起好步，对我市今后五年经济社会发展至关重要。我们要切实做好各项工作，努力实现“十三五”开门红。

今年经济社会发展主要预期目标建议为：地区生产总值增长8.5%以上，一般公共预算收入增长5%以上，规模以上固定资产投资增长12%，社会消费品零售总额增长10%，进出口总额增长10%以上，城镇和农村常住居民人均可支配收入分别增长8.5%以上和10%，居民消费价格涨幅控制在3%以内，城镇登记失业率控制在4%以内，人口自然增长率控制在7％。以内。

今年，我们将重点做好以下十个方面的工作：

（一）保持经济平稳较快增长

扩大有效投资。着力优化投资结构，改进投资方式，重点在基础设施、民生保障、新兴产业发展等领域，抓好一批亿元以上投资项目，确保投资合理增长。优化用好预算内投资，重点支持保障性安居工程、粮食、水利、节能环保、教育医疗文化等项目建设，发挥财政投资的引导撬动作用。强化以五大基础设施网络为重点的基础设施建设，加快实施地铁2号线二期、功东高速、天然气高压输配系统等一批重大项目。加大新兴产业培育和传统产业转型升级投入，力争在产业投资上取得新突破。稳定房地产投资，发挥对投资增长的支撑作用。编制“十三五”项目集群规划和三年滚动投资计划，加强重点工程、重点项目的策划、储备和滚动实施，抓紧开展禄劝至会理高速、宜良新庄水库、市艺术中心等一批项目前期工作。认真落实投资包保责任制、重点项目推进集中会办制和现场办公制，激活一批存量项目，推动一批项目尽快开工建设。

活跃市场消费。落实促进消费各项政策，引导城镇消费提档升级，挖掘农村消费潜力。着力扩大消费新供给，丰富健康、家庭、养老、护理等服务产品。稳定住房消费，提升旅游消费，培育电子信息、文化娱乐、健康养老等新的消费热点。保障市场供应，依法打击制售假冒伪劣产品、价格欺诈等行为，完善消费维权机制，维护市场竞争秩序。

强化要素保障。加快闲置土地清理和利用，完善差别化用地政策，优先保障基础设施、社会民生和重大招商引资项目用地。推广政府和社会资本合作模式，拓宽投融资渠道。抓好城市停车场、地下综合管廊、“双创”孵化等企业债券申报，做好专项建设基金项目筛选申报，争取国家、省的更大支持。健全政银企、政保企合作机制，引导金融机构加大对实体经济发展的支持力度。打好减税降费等政策组合拳，帮助企业降低成本。强化经济运行分析调度，一季度要尽快研究出台稳增长若干政策措施。

（二）加快产业转型升级

增强工业支撑作用。昆明要跨越发展，关键在产业，核心是工业。工业依然是我市经济发展的重要支撑，要更加重视工业、狠抓工业，促进全市工业转型升级、提速增效，带动三次产业融合发展。鼓励传统优势产业加大技术改造力度，引导企业兼并重组、债务重组、破产清算，积极稳妥化解过剩产能，稳住工业增长基本面。抓好昆烟打叶复烤异地技改、昆明客车搬迁技改等重点项目，开工建设浩鑫铝箔铝材深加工基地等一批亿元以上项目，力争工业投资增长12%以上。着力培育新兴产业，全力推进呈贡信息产业园建设，加快推进浪潮大数据中心、云南移动云计算中心等重大项目。新增规模以上工业企业50家、成长型中小企业40户、小微企业7000户以上。力争规模以上工业增加值增长7.5%，确保规模以上高新技术企业工业产值占工业总产值的比重达36%。推动工业园区扩量提质，完成园区基础设施投资80亿元，力争园区规模以上工业增加值增长7.5%。

促进服务业提速发展。优先发展金融、现代物流、科技及信息服务等生产性服务业，大力发展总部经济、楼宇经济。加快发展家政、养老、健康服务等生活性服务业。积极推进电子商务进农村综合示范县建设。推进一批商贸功能区、特色商业街区发展。新建、改造一批菜市场，启动“菜卡通”应用试点工作。打通居民住房消费供需通道，购租并举，增加购买存量商品房用于回迁安置比例，城镇保障性住房货币化安置率提高到50%以上。新引进金融机构2家，加快金融产业中心园区建设，金融业增加值增长10%左右。推进环滇池生态旅游文化圈规划建设，深入实施“智慧旅游”工程，鼓励发展旅游新兴业态，旅游总收入达800亿元。

提高科技创新能力。深入实施创新驱动战略，大力开展大众创业、万众创新，全社会研究与试验发展经费投入与地区生产总值之比达1.8%。新认定高新技术企业40家，新增市级重点实验室6个、工程技术研究中心11家、产业技术战略联盟2个、院士工作站5个，新增各级企业技术中心30家。深入推进科技创新梦想工程，认定20个创新创业孵化基地和公共服务平台。组织实施重点科技创新项目100项。年专利申请超过8500件、授权超过5000件，年发明专利授权量每百万人达155件。

（三）不断完善城市功能

强化规划统筹引领作用。优化区域空间布局，实现滇池流域经济和社会发展总体规划、城乡规划、土地利用总体规划、生态环境保护规划等“多规合一”。编制昆明城市远景发展战略规划、环滇池城市空间形态及天际线控制规划，做好重点地段城市设计，抓好盘龙江景观带、米轨铁路带规划，凸显昆明城市的特色与风貌。加快建设昆明规划馆。

推进重大基础设施建设。加快编制并出台“五网”建设规划。启动“五网”建设五年大会战，做好东格、宜石等高速公路项目前期工作，加快功东高速等项目建设，确保黄马高速建成通车。开工建设飞虎大道北段、春城路延长线西段等79条城市道路。加快地铁建设，确保3号线基本建成，1号线呈贡支线投入运营，开工建设5号线和9号线。启动5项地下综合管廊项目建设。加快昆明西公路客运枢纽等场站建设，完成昆明火车南站、东西广场及周边配套道路建设，保障沪昆、云桂高铁通车运行。配合推进长水国际机场配套设施、滇中引水工程和乌东德水电站建设。聚焦呈贡新区开发建设，实施基础设施建设三年行动计划，启动老城至呈贡快速通道建设，推进大渔立交等项目建设，完成昆玉高速王家营收费站改造提升工程，确保联大立交投入使用，加强教育、文体、医疗、商贸等配套设施建设，不断完善呈贡新区城市功能。

提高城市管理水平。实施16条断头路、19条道路、12个节点综合整治，优化道路交通组织，加快智能交通系统建设，着力缓解交通拥堵问题。倡导绿色出行，加快创建国家“公交都市”示范城市，主城公共交通机动化出行分担率达56%以上。积极推进立体停车场和停车换乘中心建设，鼓励社会单位开放内部停车场，主城新增机动车泊位2万个，着力缓解停车难问题。新建、改造城市公厕766座，着力解决如厕难问题。继续完善供电供水、排水防涝、垃圾污水收集处理等市政基础设施，完成45万户天然气置换。健全市容环境管理长效机制，持续整治占道经营、渣土泼洒、城郊结合部脏乱差等现象，着力改善市民居住环境。

提高城市经营能力。遵循市场规律，优化配置城市资源，盘活城市道路、桥梁、房屋等资源，推进广告权、冠名权等特许经营权市场化运作，建立城市资产保值增值管理机制，提高城市资产经营管理的效率和效益。

（四）切实做好“三农”工作

加快发展高原特色现代农业。稳定粮食生产，提升烤烟质量，推进蔬菜、花卉、苗木、畜牧、林下产业、农产品深加工等优势特色产业发展，农业增加值增长6%。建设云南高原特色农产品交易中心，大力发展“互联网+农业”，培育新型农业经营主体，扎实推进“一县一示范”工程，市级重点农业园区产值达25亿元，新建8座都市农庄，新增省级龙头企业8个、省级农民专业合作示范社26个，创建农业创业示范村20个。完善农业科技服务体系，加强农业技术推广，农业科技贡献率达到56%以上，主要农作物耕种收综合机械化水平达51%。

加快建设美丽乡村。加强农村公路建设，健全县乡村三级公路联动协管机制。开工建设柴石滩水库大型灌区，新建罗泊河等5件中小型水源工程。力争宜良海马箐和禄劝真金万水库工程完工。开展农村环境综合整治，70%的行政村建成生活污水处理设施。实施农村危房改造和抗震安居工程2.5万户。推进省级重点建设村、美丽乡村示范点、民族团结进步示范村、民族特色村和农业创业示范村建设，建成200个省市级美丽宜居乡村。

加快推进脱贫攻坚。完成3个、启动3个整乡推进。实施省级行政村整村推进15个、市级40个，精准扶贫示范村30个。建设宜居农房1万户以上，易地扶贫搬迁4000户。启动实施自然村公路路基改造和路面硬化工程。完成劳动力培训3万人，转移2.5万人。省、市扶贫到户贷款2亿元以上，扶持5000户以上贫困户。减少6万建档立卡贫困人口和7.5万边缘贫困人口，实现禄劝县脱贫摘帽。

（五）提升对外开放水平

加快开放平台建设。积极推进国家级蔬菜、花卉出口示范基地和跨境电子商务平台建设。争取在昆设立外汇交易中心。加快推进综合保税区建设，确保年内封关运营。争取国家内外贸结合商品市场试点。落实长江经济带通关一体化改革，推动昆明口岸与沿海城市港口通关协作，启动王家营铁路口岸申报建设。促进昆蓉欧班列稳定双向开行。办好第四届南博会等重要会展。

推动引进来走出去。认真落实与友好城市、地区签署的合作协议，推进产业、教育、扶贫、人才等领域的深度合作。创新招商引资机制，增强招商引资的精准性，发挥以商招商、产业招商等综合效应，积极承接产业转移，确保引进市外到位资金880亿元以上，实际利用外资16.5亿美元以上。支持有条件的企业到境外投资兴业，着力拓展发展空间。争取南亚东南亚国家在昆设立政府和商务办事机构，缔结国际友城1个。

（六）促进与滇中新区融合发展

完善融合发展机制。按照“省级决策领导、新区独立建制、市区融合发展”要求，建立健全工作机制，重点在发展规划、行政审批、产业布局、招商引资、基础设施、生态环保等方面，构建与滇中新区融合发展的机制，力争实现规划一张图、基础一张网、产业一条龙、市场一体化。

加强公共服务保障。依托我市公共服务设施和管理服务体系，为新区加快发展提供优质公共服务。按照属地管理原则，切实担负起社会管理责任，共同做好滇中新区的教育、文化、卫生、就业、社保、综治维稳、安全生产监管等工作。

共同推进项目建设。主动搞好协调服务，全力支持滇中新区做好重大项目的储备、申报工作，加快推进新昆嵩高速、东南绕城高速、机场北高速等重点交通基础设施项目，配合推进中石油安宁项目建设，协同推进水电气油、通信网络、地下综合管网等配套基础设施建设。

合力加快产业发展。突出昆明的服务功能，加强与滇中新区的产业协作，构建优势互补、布局合理的产业发展新格局。全力支持滇中新区依托安宁工业园区、杨林经济技术开发区、昆明空港经济区等重点园区，大力发展现代生物、高端装备制造、电子信息、新材料、节能环保等新兴产业，提升新区产业综合承载能力。

（七）加强生态环境建设

全力推进滇池治理。启动实施“十三五”滇池治理规划项目。继续开展草海及周边水环境提升整治，重点抓好污水管网配套建设、滇池湿地建设、入湖河道整治，完成投资20亿元以上。建立滇池流域水质监测信息系统，强化对防污、治污的管理，加强入湖河道和湿地的精细化管理，加大主城区雨污水管理调度，削减污染负荷。抓好滇池面山及“五采区”生态修复。加强水污染防治工作，推进滇池、阳宗海、牛栏江等重点流域水质持续改善。

切实加强生态保护。逐步建立生态功能区生态补偿机制和环境污染第三方治理机制，完善能源、水、土地节约集约使用制度和污染物排放管理制度。深入实施“省市联动·绿化昆明·共建春城”义务植树活动、绿色廊道及面山绿化综合整治提升工程。实施天保工程森林抚育11.5万亩、公益林建设3.6万亩、退耕还林4.7万亩、陡坡地生态治理1万亩。主城建成区新增绿地400公顷。深入推进生态县、乡（镇）、村和绿色学校、绿色社区、环境教育基地创建工作。

扎实做好节能减排。落实重点减排项目，抓好冶金、化工等重点行业节能降耗，确保完成节能减排年度目标任务。加快实施低碳、节能节水重点工程和循环经济示范县区、园区、企业建设。积极推广绿色建筑和建筑节能。加强大气污染联防联控联治，严格落实建设工地扬尘管理“六个百分百”措施，强化机动车污染防治，确保主城环境空气质量达到国家二级标准以上。

（八）深化重点领域改革

深化经济体制改革。大力发展民营经济，民营经济增加值占GDP比重达46%以上。加快滇池水务公司上市步伐。强化政府性债务管理，调整平台公司发展模式，推进投融资公司转型发展。积极稳妥发展融资登记服务机构、资本管理公司、互联网金融等新金融组织。改革预算编审体系，改进预算控制方式，完善透明预算制度，建立跨年度预算平衡机制，全面推进行政事业单位内部控制制度建设。

深化农村综合改革。加快推进农村宅基地使用权、集体建设用地使用权、农村房屋所有权确权登记颁证。建立完善农村产权流转交易市场，建成市、县、乡、村四级联动的农村产权交易服务体系。推进国家集体林业综合改革试验示范区建设。开展集体林地三权分离、林业社会化服务、公益林管理经营和林权流转机制试点。深化户籍制度改革，实行差别化落户政策。启动海口林场改革，深入推进供销社综合改革。

深化行政体制改革。全面完成县级政府机构改革，稳步推进行政类和生产经营类事业单位改革。实施机关事业单位养老保险制度改革。加快梳理公布企业投资负面清单，推进政务服务网建设。完善中介超市，扎实抓好行政审批和技术审查相分离试点工作。正式颁发统一的不动产权证书。

（九）着力促进民生改善

做好就业社保服务。实施大学生创业引领计划、农民工等人员返乡创业工程，做好职业技能培训和创业培训，新增城镇就业10万人以上，确保实名登记高校毕业生就业率达90%以上。引导在城镇稳定就业的外来务工人员参加职工社会保险，确保各类社会保险参保率保持在95%以上。新开工城镇保障性住房、棚户区改造住房1万套，基本建成2万套。

加快教育优质均衡发展。完成学前教育三年行动计划，公办和普惠性民办幼儿园在园幼儿占比达85%，每个乡镇（街道）至少建成一所公办幼儿园。确保完成盘龙、官渡、西山、安宁、晋宁、石林、禄劝义务教育基本均衡督导评估。促进高中阶段教育普及发展，加快民办教育、职业教育、特殊教育、高等教育和教育国际化发展。推进呈贡新区基础教育发展，加快市外校与北京世青国际学校合作办学，建设一批优质学校，打造优质教育资源高地。启动实施教育扶贫工程。深入推进农村薄弱学校改造、校安工程、昆明开放学院等重点项目建设。

优化卫生计生服务。加快推进分级诊疗和城市公立医院改革，扩大全科医生签约服务试点。加强公共卫生服务体系建设。健全基层医疗机构运行新机制，推进家庭医生签约制度，加强乡村医生队伍建设。加快医疗服务信息化建设和中医药事业发展。积极发展养老服务，争创国家医养结合试点示范城市。完成7个乡镇卫生院、61个村卫生室标准化建设。提升妇幼健康服务水平，全面落实一对夫妇可生育两孩政策。

促进公共文化服务均等化。推进县级“五馆”建设，提升改造乡镇（街道）文化站，启动建设基层综合性文化服务中心。加快推进龙泉古镇、飞虎队纪念馆、朱德旧居纪念馆等重点历史文化项目建设。启动聂耳大剧院、市体育中心、市足球基地项目前期工作。完成3万套广播电视“户户通”置换任务。利用昆明重机厂等老旧厂房，改造建设一批文化创意产业基地。改造老旧小区健身设施，实施“七彩云南全民健身工程”，打造城市社区“15分钟体育健身圈”。提升昆明高原国际半程马拉松赛、昆明环滇池高原自行车邀请赛等赛事品质。

营造和谐社会环境。加强法治宣传，推动全民尊法守法。发挥城乡居民自治组织的基础作用，提高治理能力，完善治理体系。加快“全国社区治理和服务创新实验区”建设，健全社区公共服务综合信息平台，提高社区综合服务管理水平。强化人民调解、行政调解、司法调解的协调联动，做好信访维稳和矛盾化解工作。深入推进平安昆明建设，依法打击暴力恐怖、涉黑涉恶、电信诈骗、网络犯罪等违法犯罪活动，打好禁毒防艾人民战争。抓好食品药品、安全生产监督管理和地质灾害防治。推进民族团结进步示范区创建工作。深入开展双拥共建，推动军民融合深度发展。

（十）强化政府自身建设

严格依法办事。坚持用法治思维和法治方式开展工作，所有行政行为都要于法有据。全面落实行政执法责任制，推进行政执法机构、职能、权限、程序、责任法定化。完善科学、民主立法机制。实现市、县、乡三级政府法律顾问全覆盖。完善重大问题决策、重要项目安排、大额资金使用决策程序。

强化权力监督。完善政务公开制度，推进决策公开、管理公开、服务公开、结果公开。加强与人大代表、政协委员以及民主党派、工商联、无党派人士和人民团体等沟通协商，认真听取意见建议，提高人大代表议案、建议和政协提案办理质量。加强新型智库建设，健全公示听证、决策咨询、专家论证等制度，提高政府决策科学化、民主化、法治化水平。充分发挥审计监督“免疫系统”功能，健全经济责任审计制度，实现审计监督全覆盖。

持续改进政风。巩固党的群众路线教育实践活动、“三严三实”和“忠诚干净担当”专题教育成果，坚决纠正“四风”，推进作风建设常态化，“三公”经费只减不增。全面完成党政机关公务用车改革。加强干部队伍建设，完善奖惩机制，集中整治不作为、乱作为等突出问题，营造想干事、能干事、干成事的良好氛围，让干事的同志有位置、有平台、有作为、有环境。严格执行廉洁自律准则和纪律处分条例，从严落实党风廉政建设主体责任和“一岗双责”，坚决惩治腐败，以反腐倡廉的实际成效取信于民。

各位代表!宏伟的蓝图已经绘就，前进的号角已经吹响。蓝图再好，关键是要干出实效，关键是要发扬钉钉子精神，锲而不舍，狠抓落实，涓涓细流汇成江河，件件实事合成大事。让我们紧密团结在以习近平同志为总书记的党中央周围，在省委、省政府和市委的坚强领导下，坚定信心、振奋精神，敢于担当、奋力拼搏，为实现昆明经济社会跨越发展、率先在全省全面建成小康社会而努力奋斗！
